# Determining the Predictors of Self-Management Behaviors in Patients With Type 2 Diabetes: An Application of Socio-Ecological Approach

**DOI:** 10.3389/fpubh.2022.820238

**Published:** 2022-04-08

**Authors:** Aghil Habibi Soola, Mahnaz Davari, Hamed Rezakhani Moghaddam

**Affiliations:** ^1^Department of Nursing, Ardabil University of Medical Sciences, Ardabil, Iran; ^2^Department of Public Health, Khalkhal University of Medical Sciences, Khalkhal, Iran

**Keywords:** predictors, complications, diabetic patients, ecological approach, self-management

## Abstract

**Background:**

Type 2 diabetes complications are responsible for 2% of hospital emergency visits. Self-management practices are one of the most essential approaches to control type 2 diabetes. The goal of this study was to use an ecological approach to investigate the predictors of self-management behaviors in diabetes patients referred to the emergency department in Ardabil in 2020.

**Methods:**

In this cross-sectional study, 273 individuals with type 2 diabetes who were sent to the emergency department of Imam Khomeini Educational and Medical Center in Ardabil were included using the available sample method. Demographic information questionnaires, including the Diabetes Distress Screening Tool (DDS2), General Diabetes Knowledge (DKT2), Diabetes Empowerment Questionnaire (DES-SF), Patient Health Questionnaire 9 (PHQ 9), Beliefs to Treatment Effectiveness Scale (BTES), Diabetes Self-Efficacy Scale (DSES), Chronic Illness Resources Survey (CIRS), Situational Effects Questionnaire, and Diabetes self-management support (DSMS), were all used to collect data. The independent *t*-test, one-way ANOVA, Pearson correlation coefficient, and multiple regression were used to analyze the data.

**Results:**

The results of the study showed that in the framework of ecological approach, predictors of self-management behaviors at the four levels are as follows: at the individual level—gender (*p* = 0.025), education (*p* = 0.002), duration of diabetes (*p* = 0.38), having a glucometer at home (*p* < 0.001), diabetes empowerment (*p* < 0.001), personal support (*p* = 0.002), and self-efficacy (*p* = 0.047); at the interpersonal level—the main health support (*p* < 0.001), membership in social networks (*p* < 0.005), family/friends support (*p* < 0.001), and neighborhoods support (*p* < 0.001); at the group and organizational level—organizational support (*p* = 0.013); at the community and policy level—the impact of mass media in health (*p* < 0.001) and situational influence (*p* < 0.001).

**Conclusion:**

The impact of non-individual levels, such as the environment, on a person's decision to manage diabetes is crucial. Diabetes management necessitates a significant amount of effort, which involves maintaining the health of diabetes patients and the community while also minimizing emergency department workload.

## Introduction

Diabetes affected 463 mi*l*lion people in 2019 according to the International Diabetes Federation. Diabetes claimed the lives of 4.2 million people worldwide between the ages of 20 and 79 in the same year (1). Type 2 diabetes has the highest prevalence of diabetes (90%) and is responsible for hundreds of billions of dollars in annual economic losses (2). Because the prevalence of type 2 diabetes is influenced by the environment, the rate of mortality and complications varies by geographic area (3). According to the WHO's six divisions, Iran is a member of the Eastern Mediterranean region, which ranks third among the 22 member nations in the number of people aged 20–79 who have diabetes. In Iran, 32,414 people died as a result of the disease's consequences in 2017 (4). Internal emergencies at the hospital include uncontrolled, early, and late consequences of type 2 diabetes.

Any hospital's emergency department (ED) is the beating heart of the facility (5). The overcrowding of patients in this ward can cause service to be disrupted. Congestion in emergency rooms is becoming a public health issue (6). According to some studies, ED congestion leads to delayed treatment, greater mortality, and longer hospital admissions (7). Type 2 diabetics in the United States have more than double the number of emergency department visits compared to other patients. On the other hand, referral to the emergency room is linked to higher medical costs. As a result, frequent ED visits might add up to a large amount of the healthcare system's total cost (8). Previous research from around the world has found a link between diabetic referral to the ED emergency room and poor diabetes control (9). Self-care and self-management are two approaches to control diabetes (10).

Self-management is a proactive and operational approach in which the patient takes the lead. Diabetes self-management is defined as a collection of actions that patients engage in on a daily basis to achieve diabetes control (11). Self-management variables in diabetes include incorporating daily life activities, such as physical activity, nutritional behaviors, blood sugar monitoring, making daily plans related to health and illness, and interpersonal communication with influential people in the field of health and illness (12). Identifying the factors that influence self-management is one of the most difficult tasks in the profession. Recognizing the psychosocial aspects that affect self-management in these individuals can help with educational planning and ensure that these programs are successful (13).

Health educators must be aware of the elements that influence learning in order to succeed in changing or consolidating healthy behavior, and theories play a role in this process. Theories provide an appropriate foundation for effective behavior intervention (10). The shortcomings of current health promotion models highlight the need for a more comprehensive strategy, which emphasizes the role of social circumstances (14). Ecological models in behavioral sciences and public health are concerned with the nature of people's interactions with their physical, cultural, and social environments (10). Health and wellbeing refer to a scenario in which one's health is thought to be influenced by a variety of factors in both the physical and social environments (e.g., as for physical ones, geography, architecture, and technology, and as for social ones, culture, economics, and politics). Individuals and groups' health is also influenced by several individual traits, such as genetic variables, psychological predispositions, and behavioral patterns, in addition to environmental factors. As a result, efforts to improve human health should be founded on an understanding of the complex interactions between various environmental and individual elements, rather than analyses that just focus on environmental, biological, or behavioral components. The socio-ecological approach assumes that increasing the effectiveness of health promotion programs (such as family members working to improve their health practices, managers of corporate health organizations shape, public health policies, and authorities overseeing public health services) can be accomplished by coordinating individuals and groups working at various levels (14). Environments and regulations that support healthy choices are expected to increase health habits (11).

The use of a socio-ecological approach as a foundation for planning and understanding the predictors of self-management behaviors in diabetic patients is emphasized in particular. As previously stated, ecological self-management necessitates access to a variety of resources (10). “Intrapersonal factors” (psychological, knowledge, skills, beliefs, attitudes, etc.), “interpersonal factors” (family friend, neighbors, etc.), “group and organization factors” (work environment, school, mosques, etc.), and “community and policy-making factors” are the four central principles of the ecological approach to diabetes self-management (health insurance support, screening program, etc.), and these categories are not mutually exclusive and unrelated (15). Individuals' skills and choices are combined with the services and support they receive in the ecological approach to diabetes self-management (10).

The development of a comprehensive pattern for identifying predictors of diabetic self-management behaviors can be used in future studies to plan for the correction or improvement of predictors of diabetic patients' self-management behaviors. Such development can also help in reducing the number of ED patients and improve the quality of care in this ward by lowering the number of referrals to the ED, improving the health of diabetic patients by lowering short- and long-term health complications, and lowering the impact on health-care costs. Few works of research have found a thorough pattern of predictors of diabetic self-management practices. The goal of this study was to use a socio-ecological approach to investigate the predictors of self-management behaviors in diabetes patients referred to the emergency department of Imam Khomeini Hospital in Ardabil.

## Methods

In this cross-sectional study, 273 patients with type 2 diabetes who were referred to the Imam Khomeini Educational and Medical Center in Ardabil in 2019 and were followed up on within 2 months were included in the study using a convenience sampling method. The number of samples was calculated using Cohen's formula for calculating sample size, as well as the acceptable error value in estimating the average, which was equal to 2 (*Z*: 1.96 and *SD*: 16.82). There was a total of 273 diabetic patients who were tested. Adults over the age of 18, had the ability to communicate, and were willing to participate in the study were all required to be included in the study. We excluded those patients with diagnosed mental and psychological disorders such as mood disorders and anxiety before being diagnosed with diabetes, as well as those suffering from a severe psychological illness after being diagnosed with diabetes and gestational diabetes.

After the visit, primary care, and hemodynamic stabilization, diabetic patients who were referred to the emergency department completed the questionnaires. In addition, the researcher completed the questionnaires for patients who were illiterate. Patients' informed consent to participate in the study was obtained, and data was collected confidentially and without anonymity in accordance with ethical principles. The Diabetes Distress Screening Tool (DDS2), General Diabetes Knowledge (DKT2), Diabetes Empowerment (DES-SF), Patient Health (PHQ 9), Horizon, Diabetes Self-Efficacy Scale (DSES), Social Support (Chronic Illness Resources Survey; CIRS), situational impacts, and Diabetes Self-Management Questionnaire (DSMS) were used to collect data.

Demographic and disease-related information of the subjects included the following: age, sex, place of residence, marital status, economic status, level of education, family composition, type of treatment, type of insurance, employment status, family history of diabetes, complications of the disease, treatment method (oral or injection), number of emergency visits in the last 12 months, number of emergency calls in the last 12 months, membership in social groups, main sponsor, use of mass media for self-management, use of means of transportation general for self-management, number of years spent with diabetes, and having a glucometer at home.

### Diabetes Distress Screening Questionnaire

Short clinical tools with simple scoring are required for rapid screening of distress in diabetic patients. According to studies, DDS2 is a primary screening tool for assessing the specific distress of diabetes and it has a high level of accuracy (96.7%). Patients were asked to rate the issues that had caused them distress in the previous month using two 6-level questions in this tool. The average total score was calculated, and if the items' average score was 3, then it indicated the average level of diabetes distress. The questionnaire's Cronbach's alpha in the main study was 0.73 (12).

### Chronic Illness Resources Survey

With 22 questions, the CIRS (16) examines the following: personal support (3 questions with a score range of 3–15), friends/family/environment (7 questions with a score range of 7–35), policies/media (3 questions with a score range of 3–15), health-related organizations (3 questions with a score range of 3–15), workplace support (3 questions with a score range of 3–15), health-related providers (3 questions with a score range of 3–15), health-related organizations (3 questions with a score range of 3–15), and health-related providers (3 questions with a score range of 3–15). The questionnaire's Cronbach's alpha was 0.81 in the Nowruz study (17).

### Situational Effects Questionnaire

The statements of a diabetic patient about the effects of the environment on people with diabetes that increase or decrease a person's commitment to diabetes self-management are evaluated using this 17-question questionnaire. Healthy eating, physical activity, drug adherence, blood sugar testing, and foot care are just a few of the topics covered. The main study had a Cronbach's alpha of 0.82 (11).

### Diabetes General Knowledge Questionnaire

This questionnaire has 14 items and is an updated version of General Diabetes Knowledge. It examines patient awareness of basic nutrition and foot care. The Cronbach's alpha for this questionnaire was 0.69, and it was utilized in Iran (18).

### Diabetes Empowerment Questionnaire (DES-SF)

The summary Chinese version of the empowerment tool includes 8 items. A higher-than-average DES-SF score indicates a higher capability, while a lower-than-average score indicates a lesser capability. The questionnaire's Cronbach's alpha was 0.85 in Young's study (19).

### Patient Health Questionnaire 9

A patient health model for depression is the 9-item Patient Health Questionnaire. It has been translated into Persian and culturally tailored for diabetics in Iran (20). This questionnaire consists of nine questions, each of which has a response relating to the previous 2 weeks. From 0 to 27, the overall score ranges from 0 to 27. Moderate depression is defined as a score of 15, while severe depression is defined as a score of 20. In Iran, Cronbach's alpha was 0.88 (21).

### Beliefs to Treatment Effectiveness Scale

Based on multiple similar quizzes, Rahimian Booger came up with this expression. The patient's belief in the efficiency of self-management practices in controlling diabetes and reducing complications was examined in this questionnaire, which contains nine items. The first four questions looked at whether people believe diabetic self-management activities are important for diabetes control. The questionnaire's final five questions probed respondents' perceptions about how significant diabetes self-management activities are in preventing diabetes complications. This tool uses an 11-point Likert scale that ranges from 0% (never) to 100% (always). Cronbach's alpha was 0.94 in the main study (22).

### Diabetes Self-Efficacy Scale

The DSES questionnaire has eight questions (11) that are used to assess self-efficacy in performing parts of diabetes self-management, such as diet (items 1, 2, and 3), exercise (items 4 and 5), and sugar drop or increase management. Depending on the drug, blood (item 6) was used (item 7, 8). On a 10-point Likert scale of 1, respondents were asked to rate their confidence in managing diabetes. The questionnaire has a score range of 8–80, with a higher score indicating a higher level of self-efficacy. Cronbach's alpha for this questionnaire was 0.98 in Tol's study in Iran (21).

### Diabetes Self-Management Support

There are 35 questions on the Lane DSMS Questionnaire (23). This questionnaire has 32 items in a 4-choice format in the Iranian version (24). The lowest and highest scores were 32 and 128, respectively. More diabetes self-management activity is associated with a better score. Healthy eating and lifestyle (5 questions), self-regulation (7 questions), adherence to medication regimen (3 questions), disease compliance (5 questions), blood sugar monitoring (3 questions), interaction with doctors and health care providers (6 questions), and interaction with important people (3 questions) are the seven subscales of this tool (3 questions). In the Tahmasebi study, Cronbach's alpha was 0.92.

After completing the questionnaires, the data were entered into the IBM Statistical Package for the Social Sciences (SPSS 22) software and analyzed. All study variables were analyzed using descriptive statistics (mean, *SD*, frequency, and percentage). The relationship and correlation between the independent variables of the four categories of socio-ecological approach with self-management were investigated using the independent *t*-test, one-way ANOVA, and Pearson correlation coefficient. The predictive value of each of the self-management factors was then determined using multiple regression analysis.

The Kolmogorov-Smirnov test was used to determine data normality, and the study data were found to be normal. To study the relationship and correlation between the independent variables of the four categories of ecological approach with self-management, descriptive statistics, independent *t*-test, one-way ANOVA, and Pearson correlation coefficient were utilized.

## Results

In the individual factors, the mean age of participants was (61.33 ± 9.67) and the duration of diabetes was (6.69 ± 1.69). A total of 165 participants (60.4%) were men and 192 (70.4%) were married. Among the participants, 162 (59.3%) were economically dependent, 127 (45.6 %) were illiterate while 162 (59.3%) were unemployed, 135 (49.4%) were receiving drug treatment, 157 (57.9%) reported complications of diabetes, and 137 (50.2%) did not have a glucometer at home. In the past year, 102 of the participants (37.4%) have been to the emergency department at least once, and 164 (60.1%) have not reported any emergency medical services (EMS) contact. Furthermore, 160 of the participants (58.6%) reported better diabetes knowledge and 164 (60.1%) had high diabetes empowerment. Diabetes distress was reported in 245 (89.7%) participants and depression in 132 (48.4%) participants. The mean of “belief in treatment effectiveness,” “self-efficacy,” and “personal support” of the participants were (76.05 ± 11.25), (52.19 ± 10.75), and (10.16 ± 2.31), respectively.

Diabetes self-management with individual variables; gender, age, level of education, type of treatment, employment status, duration of diabetes, having a glucometer at home, refer to emergency in the last 12 months, emergency medical call within the last 12 months, diabetes distress, diabetes knowledge, patient ability, depression, belief in the effectiveness, personal support, and self-efficacy all had a statistically significant relationship (p < 0.05).

In the interpersonal factors; 159 (58.2%) of participants stated that they live with their families. In 160 (58.6%) of the participants, their child was the main health supporter. One hundred and seven (62.3%) of them said they were members of social media sites. The averages for “family/friends support” and “neighborhood support” were, respectively (10.23 ± 2.55) and (7.74 ± 2.85). Family composition, major health supporter, family/friends support, and neighborhood support all had a statistically significant relationship with diabetes self-management (*p* < 0.05).

In the “group and organization factors”, the study participants' mean scores for “health care support,” “organizational support,” and “workplace support” were (3.02 ± 0.95), (1.35 ± 0.53), and (1.70 ± 0.64), respectively. Health care support, organizational support, and workplace support all demonstrated statistically significant relationships with diabetes self-management (*p* < 0.05).

In the “community and policy factors”, 205 people (75.1%) said they lived in the city. The results for “the impact of mass media on health” and “the impact of public transportation on health” were 170 (62.3%) positive and 161 (95.6%) negative, respectively. Social security insurance supported 78 of the participants. A “medium” situational influence was noted by 193 of the participants. The average “policy support” score was 8.15 ± 2.48. Type of insurance, situational influence, and policy support all had statistically significant relationships with diabetes self-management (*p* < 0.05; Table 1).

**Table 1:** Descriptive table of independent variables and their relationship with self-management.

	**Variables**	**Number (%) or mean (SD)**	**The relationship between independent variables and diabetes self-management**	
			**Mean (SD)**	**Statistical tests and *P*-value**
	**Age**	61.33 ± 9.67		*r* = 0.218 *p* < 0.001
	**Gender**			
	male	108 (39.6)	82.49 ±12.37	*t* = 3.98 *P* < 0.001
	female	165 (60.4)	88.18 ±10.13	
	**Marital status**			
	Single or divorced	11(4)	87.45 ± 19.63	*F* = 3.39 *P* = 0.35
	Married	192 (70.4)	85.71 ± 11.09	
	Widowed	70 (25.6)	81.64 ±12	
	**Level of education**			
	Not read and write	127(45.6)	81.16 ± 11.02	*F* = 8.18 *P* < 0.001
	Read and write	42 (15.4)	87.23 ± 9.11	
	Elementary school—Junior high school attended	64 (23.3)	89 ± 6.4	
	Senior high school -College/above	40 (14.7)	86.67 ± 15.1	
	**Type of economy**			
	Independent	111 (40.7)	85.79 ± 12.91	*t* = 1.21 *P* = 0.22
	Dependent	162 (59.3)	84.02 ± 11.05	
	**Occupational status**			
	Unemployed	162 (59.3)	84.01 ± 11.11	*F* = 3.22 *P* = 0.04
	Employed	77 (28.2)	84.16 ± 10.88	
	Retired	33 (12.5)	89.52 ± 16	
	**Time since diabetes diagnosis year**	6.69 ± 1.69		*r* = 0.231 *p* < 0.001
**Individual factors**	**Type of treatment**			
	Drug	135 (49.4)	89.85 ± 11.16	*F* = 3.44 *P* = 0.03
	Insulin	69 (25.3)	85.69 ± 11.30	
	Both drug and insulin	69 (25.3)	81.53 ± 12.38	
	**Diabetes complications**			
	Yes	157 (57.9)	84.13 ± 12.01	*t* = 0.99 *P* = 0.31
	No	115 (42.1)	85.58 ± 11.62	
	**Have glucometer at home**			
	Yes	136 (49.8)	87.84 ± 12.26	*t* = 4.45 *P* < 0.001
	No	137 (50.2)	81.66 ± 10.60	
	**Refer to emergency in the last 12 months**			
	1 time	102 (37.4)	86.73 ± 12.27	*F* = 7.60 *P* < 0.001
	2 times	91 (33.3)	86.95 ± 10.76	
	3 times	51 (18.7)	80.50 ± 11.82	
	4 times and more	29 (10.6)	78.24 ± 9.24	
	**Emergency medical call with in the last 12 months**			
	No phone call	164 (60.1)	86.40 ± 11.46	*F* = 15.17 *P* < 0.001
	1 time	70 (25.6)	85.98 ± 11.05	
	2 times and more	39 (14.3)	75.53 ± 10.90	
	**Diabetes knowledge**			
	Have better knowledge	160 (58.6)	87.91 ± 12.15	*t* = 5.54 *P* < 0.001
	Have less knowledge	113 (41.4)	24.50 ± 9.84	
	**Diabetes distress**			
	No diabetes distress	28 (10.3)	96.82 ± 10.50	*t* = 6.05 *P* < 0.001
	Diabetes distress	245 (89.7)	36.83 ± 11.21	
	**Depression status**			
	No depression	132 (48.4)	87.87 ± 11.39	*F* = 10.93 *P* < 0.001
	Minor depression	117 (42.9)	82.55 ± 11.87	
	Major depression	24 (8.7)	78.20 ± 9.26	
	**Diabetes empowerment**			
	High empowerment	164 (61.1)	90.75 ± 8.88	*t* = 13.11 *P* < 0.001
	Low empowerment	109 (39.9)	24.80 ± 9.84	
	**Diabetes self-efficacy**	52.19 ± 10.75		*r* = 0.228 *p* < 0.01
	**Belief in effectiveness**	70.05 ± 11.25		*r* = 0.535 *p* < 0.01
	**Personal support**	10.16 ± 2.31		*r* = 0.513 *p* < 0.01
**Interpersonal factors**	**Family composition**			
	With family	159 (58.2)	84.16 ± 10.86	*F* = 4.16 *P* = 0.007
	With wife	47 (17.2)	89.93 ± 14	
	With children	42 (15.4)	81.95 ± 13.61	
	Single	25 (9.2)	83.36 ± 7.26	
	**Main health supporter**			
	Wife	71 (26)	83.56 ± 10.79	*F* = 5.18 *P* = 0.006
	Child	160 (58.6)	83.86 ± 10.79	
	Other	42 (15.4)	90.07 ± 12.67	
	**Join social networks**			
	Yes	103 (37.7)	86.61 ± 12.18	*t* = 2.03 *P* = 0.67
	No	170 (62.3)	83.61 ± 54.11	
	**Family/friends support**	10.23 ± 2.55		*r* = 0.396 *P* < 0.001
	**Neighborhood support**	7.74 ± 2.85		*r* = 0.399 *P* < 0.001
**Group and organization factors**	**Health care support**	3.02 ± 0.95		*r* = 0.342 *P* < 0.001
	**Organizational support**	1.35 ± 0.53		*r* = 0.348 *P* < 0.001
	**Workplace support**	1.70 ± 0.64		*r* = 0.270 *P* < 0.001
**Community and policy factors**	**Location**			
	City	205 (75.1)	84.37 ± 12.12	*t* = 0.902 *P* = 0.44
	Village	68 (24.9)	85.86 ± 85.86	
	**The impact of mass media in health**			
	Yes	170 (62.3)	87.44 ± 11.75	*t* = 5.04 *P* = 0.31
	No	103 (37.7)	80.29 ± 10.66	
	**Impact of public transport in health**			
	Yes	12 (4.4)	83.66 ± 89.9	*t* = 0.32 *P* = 0.19
	No	161 (95.6)	84.79 ± 11.95	
	**Type of insurance**			
	No insurance	41 (15)	84.65 ± 11	*F* = 2.71 *P* = 0.02
	health Service	56 (20.5)	87.93 ± 13.51	
	Social Security	78 (28.6)	81.93 ± 10.94	
	Health	35 (12/8)	82.77 ± 12.30	
	rural	7 (2.6)	93 ± 13.17	
	Special companies	56 (20.5)	85.75 ± 10.51	
	**Situational influence**			
	Low	40 (14.7)	79.17 ± 87	*F* = 58.27 *P* < 0.001
	medium	193 (70.7)	82.25 ± 10.03	
	Much	40 (14.7)	102.33 ± 5.35	
	**Policy support**	8.15 ± 2.48		*r* = 0.280 *P* < 0.001

The participants' self-management score averaged 84.7 ± 11.85 (minimum: 55—maximum: 115 and range; 32–120). “Adherence to suggested regimen” (3.1 ± 0.57) was the highest and “Interaction with health experts” (2.25 ± 56.5) was the lowest of the self-management behaviors subscales (Table 2).

**Table 2:** Descriptive table of diabetes self-management (*n* = 273).

**Variables**	**Mean (SD)**		**Range**
Diabetes self-management total	84.74 ± 11.85	Min 55 Max 115	32–120
Nutrition and healthy lifestyle	2.79 ± 0.60		1–4
Illness adaptation	2.34 ± 0.57		1–4
Self- regulation	2.77 ± 0.40		1–4
Interaction with health professionals	2.25 ± 0.56		1–4
Interaction with significant others	2.29 ± 0.68		1–4
Self- monitoring blood glucose	2.31 ± 0.71		1–4
Adherence to recommended regimen	3.1 ± 0.57		1–4

The results obtained from the multiple regression model for predicting self-management behaviors of diabetic patients who were referred to the emergency department according to the ecological approach are shown in Table 3. The results showed that the variables of gender, level of education, occupational status, having a glucometer at home, diabetes distress, diabetes empowerment, belief in the effectiveness, and diabetes self-efficacy among the “individual factors”, variables of main health supporter, joint social networks, family/friends support, and neighborhood support among the “interpersonal factors”, variable of organizational support as the only predictor among the “group and organization factors”, and variables of situational influence and the impact of mass media in health among the “community and policy factors” were significant predictors of self-management behaviors (*F* = 19.45, *P* < 0.001, *R* = 0.51).

**Table 3:** Regression analysis of independent variables with self-management of the study population.

	**Variables**	***b***	**Std**	**Beta**	***t***	***p***
Individual	Age	0.072	−0.030	−0.025	−0.422	0.672
	Gender	1.235	2.783	0.115	2.254	0.025
	marital status	1.006	0.340	0.014	0.337	0.763
	Educational Status	0.528	1.666	0.159	3.157	0.002
	Type of economy	1.38	0.537	0.022	0.395	0.693
	Occupational Status	0.146	0.315	0.096	2.156	0.032
	Time since diabetes diagnosis (year)	0.855	0.751	0.045	0.879	0.380
	Type of treatment	0.85	−0.081	−0.001	−0.031	0.975
	Diabetes Complications	1.009	0.511	0.021	0.506	0.613
	Have glucometer at home	0.968	−3.403	−0.144	−3.516	<0.001
	Refer to Emergency in the last 12 months	0.546	0.789	0.069	1.444	0.150
	Emergency medical call with in the last 12 months	0.611	−0.928	−0.076	−1.159	0.130
	Diabetes knowledge	0.276	0.400	0.064	1.451	0.148
	Diabetes distress	0.096	0.987	0.486	10.275	<0.001
	Depression status	0.085	−0.167	−0.083	−1.963	0.050
	Diabetes empowerment	0.048	−0.002	0.002	−0.049	<0.001
	Diabetes self-efficacy	0.236	0.749	0.146	3.171	0.002
	Belief in effectiveness	0.056	0.112	0.102	1.997	0.047
	Personal support	0.514	−0.016	−0.006	−0.120	0.905
Interpersonal	Family composition	0.781	−1.155	−0.079	−1.479	0.140
	main health supporter	0.990	2.788	−0.150	2.816	0.017
	Join social networks	1.284	−2.323	−0.095	−1.810	0.005
	Family/friends support	0.267	1.282	0.276	4.809	<0.001
	Neighborhood support	0.240	1.197	0.288	4.989	<0.001
Group and organization factors	health care support	0.584	0.0748	0.142	1.282	0.204
	Organizational support	0.862	2/190	0.320	2.542	0.013
	Work support	0.753	0/413	0.071	0.549	0.585
Community and policy	Location	1.508	1/075	0.039	0.715	0.47
	The impact of mass media in health	1.262	−4.719	−0.193	−3.74	<0.001
	Impact of public transport in health	1.914	1.235	0.021	0.424	0.67
	Type of insurance	0.396	0.719	0.102	0.816	0.70
	Situational influence	1.140	9.940	0.503	8.717	<0.001
	Policy support	0.273	0.121	0.025	0.442	0.65

*R = 0.51, F = 19.45, p < 0.001*.

## Discussion

Based on a socio-ecological approach in 2020, the goal of this study was to evaluate the predictors of self-management behaviors in diabetes patients referred to the emergency department of Imam Khomeini Hospital in Ardabil. The findings suggest that in the individual dimension, diabetes ability is one of the determinants of individual factors in diabetic patient self-management. Yang et al.'s study in China (25) and Arda et al.'s study in Turkey (26) are in line with our findings. Furthermore, according to a recent study, people who succeed in becoming empowered urge other diabetic patients to improve their blood glucose management (27). Perhaps the patient's sense of responsibility and control over his or her sickness might be defined as empowerment. Patient empowerment in the individual dimension can be affected by other levels of the ecological approach such as interpersonal variables. This is based on the integration of levels in the framework of the socio-ecological approach, wherein levels can hinder or support each other (family, group in disease, etc.).

Costa determined in Brazil that self-efficacy is a crucial element in the success of self-management in chronic diseases like diabetes and that increasing self-efficacy is critical for empowering these patients (28). The results of the Kurnia study in Indonesia corroborate our findings on self-efficacy (11). On the other hand, self-efficacy can vary and decide people's behavior through time according to the study of Bandura (29). These findings support an ecological perspective in which the long-term success of self-management is dependent on the context in which it occurs. According to paragraph four of the principles of ecological perspectives in changing health behaviors, self-efficacy is probably a significant indication in changing health behaviors that might be inhibitors or inducers of particular behaviors.

In terms of educational level, our findings are similar to Costa's findings in Brazil (28). Rahimian concluded in his study that education had a favorable link with understanding health information, and that understanding information aids the self-management process (30). Given that the majority of people with diabetes who were referred to the emergency department in this study had a low level of education. Since education has been shown to predict diabetes self-management behaviors, it is likely that people with poor diabetes self-management are the most likely to have emergency diabetes.

In terms of diabetes duration, the findings of this study are similar to those of Cornia's study in Indonesia (11). Jay's research found that most patients with the quickest time to diagnosis, such as those diagnosed in the first or second year with the most serious diseases, have been referred to the emergency room. This could be due to the fact that patients with a longer history of diabetes have superior diabetes management abilities and have been able to adapt their lifestyle to their illness over time (31). Diet adherence improves with the number of years a person has had diabetes, according to the Arda et al. study (26). However, several works of research have discovered no link between diabetes duration and self-management habits (32). From an ecological standpoint, the long-term success of self-management is determined by the circumstances in which the individual exists. As a result, various levels of assistance should be considered when planning for behavior change in these individuals throughout time. Patients' emergency referrals are likely to be reduced in the future if doctors communicate with them and increase their education, especially in the early years.

The majority of the participants in this study were men with lower levels of self-control than women. This finding is in line with research conducted in Cornia, Indonesia (11) and Diriba, Ethiopia (33). It is claimed that because clinics are overcrowded and men who are busier squander a lot of time, these people visit clinics less and are obliged to go to the emergency room in an emergency. This results in giving some help at work or adjusting the working hours of clinics for persons with diabetes, which falls under the “group and organization factors” facet of ecological theory. It may assist these patients in developing self-management skills.

Our findings on individual support are in line with Yang et al.'s Taiwanese research (19). According to the framework of an ecological approach, Jiamjarasrangi's findings also reveal that “individual support” and “neighborhood support” predict self-management among type 2 diabetes patients in Thailand's metropolitan areas. Individual support is negatively associated with neighborhood support, according to the findings of this study (34).

Our findings on having a glucometer at home were similar to those of Tirunesh (35) who found that knowing glucose levels was significantly associated with good self-care practice. This study found that those who had fasting glucose levels 2.7 times higher than their counterparts were associated with good self-care performance (36). According to the socio-ecological approach, obstacles in front of the glucometer, such as financial problems, easier preparation of glucometer kits, and training on how to use, motivation, and patient understanding of the need for this device for better self-regulation and self-management should be considered for macro and micro planning to promote the health and care of these patients.

Support from family and friends predicts diabetic patients' self-management actions in the emergency department. The findings of our research agree with those of Diriba in Ethiopia (33). Positive family relationships empower people and make it easier to cope with disease (27). Self-management might be hampered by the patient's family, or it can be facilitated by the patient's family. Some of them, for example, provide healthy meals for the diabetes patient, while others prepare bad meals for the diabetic patient. As a result, the involvement of family and friends in the self-management of diabetic patients is especially vital for those patients who have a strong cultural focus on family bonds.

According to the findings of Bouldin's study, the primary health supporter, such as a family member or friend (a close relative of the patient), plays a critical role in diabetes self-management (37). According to Lee, less perceived support was linked to higher diabetes discomfort, lower self-efficacy, and, most crucially, lower individual support (12). Friends should be transformed into practice by modeling and receiving support from family after learning self-care behavior in diabetic patients from health care providers. The support of the primary health care provider in patients is likely to have good effects on various beliefs, attitudes, and behaviors linked to self-management by categorizing the factors impacting self-management in an ecological setting. This is consistent with the study's findings and demonstrates the critical function of the main health supporter (one of the patient's relatives) in achieving self-management.

Neighborhood support had a substantial link with diabetic self-management and was a predictor of diabetes self-management, according to the findings. As previously indicated, Jiamjarasrangsi's study in Taiwan discovered that “neighborhood support, neighborhood” is a predictor of self-management among type 2 diabetic patients in Thailand's urban region (34). This aspect can be evaluated from an ecological standpoint by looking at the prevailing culture in society. Our findings are also in line with a Vassilev study, which found that older adults with diabetes had a limited ability to use social media (38). Given the average age of the participants in this study, it's safe to assume that people in our study had a limited opportunity to join social networks. However, the religious culture of the society in relation to the participation of women and the elderly in various social networks may have played a role in this outcome.

Our findings in terms of organizational support are similar to those of Dao (39). When compared to other low-income community organizations, the health care organization is regarded to have the highest rate of perceived support. This could be because patients are typically the main source of healthcare employees. Know how to get help with your health. Other community organizations, on the other hand, do not have substantial roles and duties in health care (34).

The media's impact on health has been identified as a predictor of diabetic self-management activities. This conclusion was in contrast to Noroozi's findings in Iran, which revealed that mass media, such as radio and television, have a favorable effect on women's participation in physical exercise and are an essential source of encouragement (17, 40). This is due to the fact that the majority of the participants in this study were retired or housewives who listened to the radio and television, particularly the provincial media in the local dialect.

Based on the socio-ecological approach, community-based interventions in the health of diabetic patients have shown that focusing on community members rather than specific individuals is cost-effective and promising, and has the potential to alleviate the burden of diabetes (41). Policies, media, and service delivery organizations and systems, in addition to personal adjustment, are powerful support factors for health-promoting behaviors. In line with the Noroozi et al. study (17), policy support did not predict diabetic self-management behavior in our study. On the other hand, the scarcity of research in this area has made it difficult to make conclusions, and additional research is needed to adequately assess the impact of these support resources.

In emergency diabetic patients, another predictive situational effect of diabetes self-management was discovered. Our findings are in line with those of Kurnia et al. in Indonesia (11). According to Dao et al.'s research, cultural impediments to eating a healthy diet exist among Eastern cultures at the communal level (39). Pender claims that environmental factors have a direct impact on behavior in his health promotion hypothesis (11). Self-management habits in diabetes patients will improve as a result of modifying the environment around them.

## Limitations

One of the study's limitations was the lack of similar internal and external studies in the field of predictors of diabetes self-management using an ecological approach, which makes it difficult to draw conclusions in this field and highlights the need for research on chronic disease patients.

Furthermore, because the study was limited to the Imam Khomeini Medical Center in Ardabil, which is the city's only internal emergency department, caution should be given in extrapolating the findings to other diabetes patients. Moreover, because data was acquired using self-report tools and the nurse collected it, data may be prone to social utility bias, and the use of several questionnaires may have resulted in erroneous patient responses. Finally, because this was a cross-sectional study, it may not be decisive in the long run.

## Conclusion

Regrettably, diabetes patients have the fewest sources of assistance in factors of organizational, community, and policy considerations. Individual characteristics, followed by interpersonal factors, are the most important determinants of self-management activities in these patients. The impact of other factors, including the environment, on a person's ability to manage diabetes is critical. According to the ecological approach, because many levels interact with each other, strategies that focus on one component to improving patient self-management may not be adequate to create a lasting influence on self-management behaviors. Multi-level interventions should also be employed to offer environmental circumstances with realistic and specific methods of each culture for the right and ongoing treatment of people's diabetes for deliberate planning for change at each level. As a result, success in diabetes self-management necessitates the complete participation of the patient, significant others in their life, linked organizations, their community, and policymakers, all of them ensuring the promotion of diabetes patients and the community.

## Data Availability Statement

The raw data supporting the conclusions of this article will be made available by the authors, without undue reservation.

## Ethics Statement

The protocol of this study was approved by the Research Ethics Committee, Ardabil University of Medical Sciences (IR.ARUMS.REC.1398.455). Also, Ethical issues have been completely observed by the authors.

## Author Contributions

MD conceptualized, designed, conducted and analyzed the research, which formed part of his MSc, and drafted the article. AH supervised the first authors research, inputting on the design, implementation and findings of the research, and contributed to the final article. HR co-supervised the research, inputting on the research finding and contributing to the final article, and contributed to conceptualize and design of study. All authors contributed to the article and approved the submitted version.

## Conflict of Interest

The authors declare that the research was conducted in the absence of any commercial or financial relationships that could be construed as a potential conflict of interest.

## Publisher's Note

All claims expressed in this article are solely those of the authors and do not necessarily represent those of their affiliated organizations, or those of the publisher, the editors and the reviewers. Any product that may be evaluated in this article, or claim that may be made by its manufacturer, is not guaranteed or endorsed by the publisher.
